# Physician Density by Specialty Type in Urban and Rural Counties in the US, 2010 to 2017

**DOI:** 10.1001/jamanetworkopen.2020.33994

**Published:** 2021-01-22

**Authors:** Sara R. Machado, Sahan Jayawardana, Elias Mossialos, Muthiah Vaduganathan

**Affiliations:** 1 London School of Economics and Political Science, London, United Kingdom; 2 Heart & Vascular Center, Brigham and Women’s Hospital, Harvard Medical School, Boston, Massachusetts

## Abstract

This cross-sectional study uses data from the American Medical Association Physician Masterfile to examine physician density by specialty type across metropolitan and rural US counties from 2010 to 2017.

## Introduction

Previous studies have highlighted uneven physician distribution, a key determinant of health care access, across the US.^1^ However, little is known about how these patterns have evolved over time. This study examined recent trends in physician density by specialty category across rural and urban US counties.

## Methods

For this cross-sectional study, the number of nonfederal physicians per county was collected from the American Medical Association Physician Masterfile (2010-2017). Physicians were classified as primary care physicians, medical specialists, surgical specialists, or other specialists (eMethods in the Supplement). Counties were classified as large metropolitan, medium or small metropolitan, or rural based on the Centers for Disease Control and Prevention’s (CDC) Wide-ranging Online Data for Epidemiologic Research database. Mass General Brigham deemed this study exempt from institutional review board approval and the need for informed consent because it evaluated publicly available, deidentified data. This study followed the Strengthening the Reporting of Observational Studies in Epidemiology (STROBE) reporting guideline.

The primary outcome of interest, physician density per 100 000 persons, was measured annually from 2010 to 2017. The primary comparison evaluated differences in absolute changes in physician density across types of counties during the study period. Differences in trends across 3 urban-rural subgroups were assessed using a Chow test. Mean annual trends were adjusted to account for time-updated annual county-level characteristics, collected from the American Community Survey, that may have influenced physician reimbursement, including the proportion of patients older than 65 years (as a proxy for local Medicare coverage) and median household income. All statistical testing was 2-sided with an a priori level of significance of *P* < .05. Statistical analyses were performed from June 24, 2020, to November 25, 2020, using Stata statistical software, version 15.1 (StataCorp LLC).

## Results

**Figure 1.**
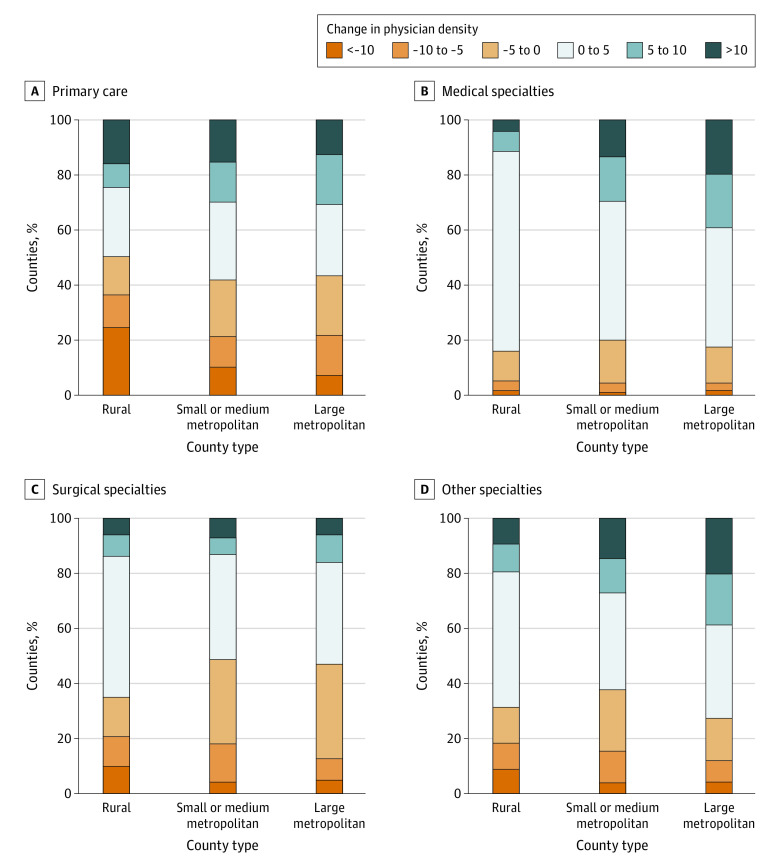
Absolute Change in Physician Density by Specialty Type From 2010 to 2017 Change in physician density was measured as absolute change in the total number of physicians per 100 000 population between 2010 and 2017 by specialty type, estimated for each type of county.

**Figure 2.**
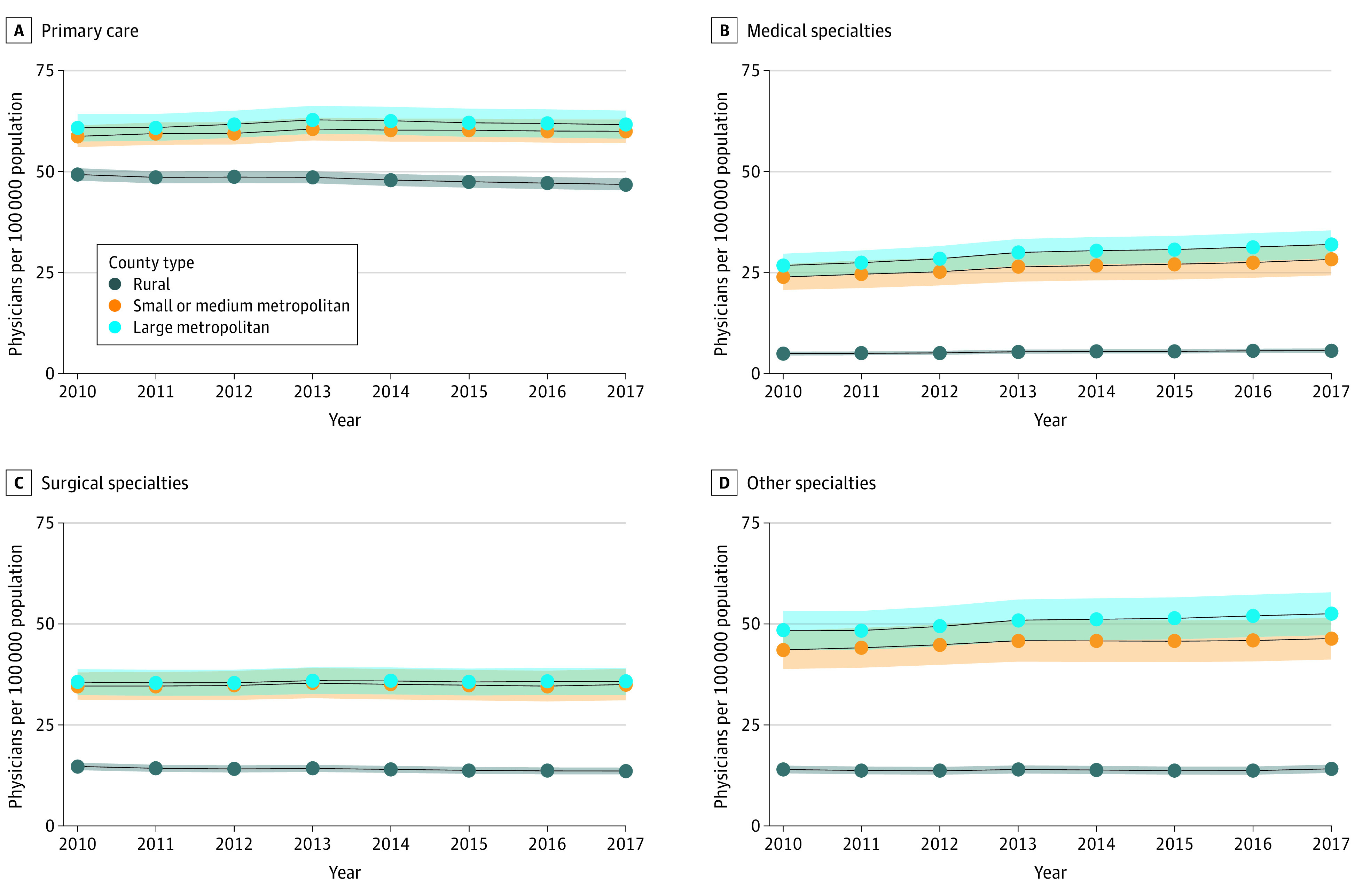
Physician Density Across Rural, Small or Medium Metropolitan, and Large Metropolitan Counties by Specialty Category From 2010 to 2017 Solid lines indicate means. Shaded areas indicate 95% CIs.

Overall, 3142 counties (436 large metropolitan, 730 small or medium metropolitan, and 1976 rural) were assessed. From 2010 to 2017, the median physician density was higher in urban counties (large metropolitan counties: 125.3 [interquartile range (IQR), 62.5-255.0] physicians per 100 000 persons; small or medium metropolitan counties: 124.3 [IQR, 50.4-215.7] physicians per 100 000 persons) compared with rural counties (59.7 [IQR, 32.0-101.1] physicians per 100 000 persons). From 2010 to 2017, overall physician density increased by a mean (SD) of 1.5 (39.0) physicians per 100 000 persons, but this varied by county type; large metropolitan counties had a mean (SD) increase of 10.0 (37.9) physicians per 100 000 persons, small or medium metropolitan counties had a mean (SD) increase of 8.8 (46.2) physicians per 100 000 persons, and rural counties had a mean (SD) decrease of 3.1 (35.5) physicians per 100 000 persons. Primary care density decreased in 189 (43.3%) of the large metropolitan counties, 305 (41.8%) of the small or medium metropolitan counties, and 994 (50.3%) of the rural counties over time (Figure 1). The mean density of primary care physicians decreased from 2010 to 2017, particularly in rural counties (Figure 2). Trends from 2010 to 2017 significantly diverged between rural counties and small, medium, or large metropolitan counties across all specialty types.

## Discussion

This cross-sectional study of the US population showed a higher density of primary care physicians and specialists in urban compared with rural counties. This rural-urban gap persisted with modest but significant divergence in recent years. The workforce of primary care physicians steadily decreased across more than half of rural counties. Aging of the rural physician workforce,^2^ which may be associated with increased clinician shortages,^3^ is expected to further be associated with decreased physician density in these areas.^4^

Physician shortages may contribute to current gaps in population health between urban and rural counties.^5,6^ This study’s data support improved incentive structures to redistribute physician resources to match the unequal health burden experienced by rural and urban populations in the US. A limitation of this study is that it relied on 1 urban-rural classification scheme proposed by the CDC in which county designations appeared to track with other health measures; however, other schema may differentially categorize counties. Physicians practicing in locations outside their county of registration introduced potential misclassification. Further research is needed to evaluate potential multifaceted policies to improve equity of physician distribution and attendant implications for access to care and overall health system quality.

## Notes

Supplement. **eMethods.** Physician Specialty Classification
