# Supplementary material for: Physician Density by Specialty Type in Urban and Rural Counties in the US, 2010 to 2017
Source: JAMA Netw Open. 2021 Jan 22;4(1):e2033994. doi: 10.1001/jamanetworkopen.2020.33994 (PMC7823223; doi:10.1001/jamanetworkopen.2020.33994)
Supplement: Supplement. — eMethods. Physician Specialty Classification [file jamanetwopen-e2033994-s001.pdf]

Supplementary Online Content

Machado SR, Jayawardana S, Mossialos E, Vaduganathan M. Physician density by specialty type in urban and rural counties in the US, 2010 to 2017. *JAMA Netw Open*. 2021;4(1):e2033994. doi:10.1001/jamanetworkopen.2020.33994

eMethods. Physician Specialty Classification

This supplementary material has been provided by the authors to give readers additional information about their work.

© 2021 Machado SR et al. *JAMA Network Open*.

eMethods. Physician Specialty Classification

Primary Care: General Family Medicine, General Practice, General Internal Medicine, and General Pediatrics.
Medical Specialties: Allergy and Immunology, Cardiovascular Disease, Dermatology, Epidemiology, Gastroenterology, Internal Medicine, Internal Medicine Subspecialties, Pediatrics, Pediatric Subspecialties, Pediatric Cardiology, Pulmonary Disease
Surgical Specialties: Colon/Rectal Surgery, General Surgery, Neurological Surgery, Obstetrics Gynecology, Obstetrics Gynecology Subspecialties, Ophthalmology, Orthopedic Surgery, Otolaryngology, Plastic Surgery, Thoracic Surgery, Urology
Other Specialties: Aerospace Medicine, Anesthesiology, Child + Adolescent Psychiatry, Diagnostic Radiology, Emergency Medicine, Forensic Pathology, General Preventive Medicine, Medical Genetics, Neurology, Nuclear Medicine, Occupational Medicine, Psychiatry, Pathology, Anatomic/Clinical, Physical Medicine/Rehabilitation, General Preventive Medicine, Radiology, Radiation Oncology, Transplantation Surgery (part of Surgical Specialties in 2000), Vascular Medicine (part of Medical Specialties in 2000), Other Specialties, Unspecified

© 2021 Machado SR et al. *JAMA Network Open*.
